# 5-Bromo-7-methyl-2-(4-methyl­phen­yl)-3-methyl­sulfinyl-1-benzofuran

**DOI:** 10.1107/S160053681204682X

**Published:** 2012-11-28

**Authors:** Hong Dae Choi, Pil Ja Seo, Uk Lee

**Affiliations:** a Department of Chemistry, Dongeui University, San 24 Kaya-dong, Busanjin-gu, Busan 614-714, Republic of Korea; b Department of Chemistry, Pukyong National University, 599-1 Daeyeon 3-dong, Nam-gu, Busan 608-737, Republic of Korea

## Abstract

In the title compound, C_17_H_15_BrO_2_S, the 4-methyl­phenyl ring makes a dihedral angle of 14.46 (5)° with the mean plane [r.m.s. deviation = 0.005 (1) Å] of the benzofuran fragment. In the crystal, mol­ecules are linked by pairs of Br⋯O contacts [3.151 (2) Å] into centrosymmetric dimers.

## Related literature

For background information and the crystal structures of related compounds, see: Choi *et al.* (2010 ▶); Seo *et al.* (2009 ▶). For a review of halogen bonding, see: Politzer *et al.* (2007 ▶).
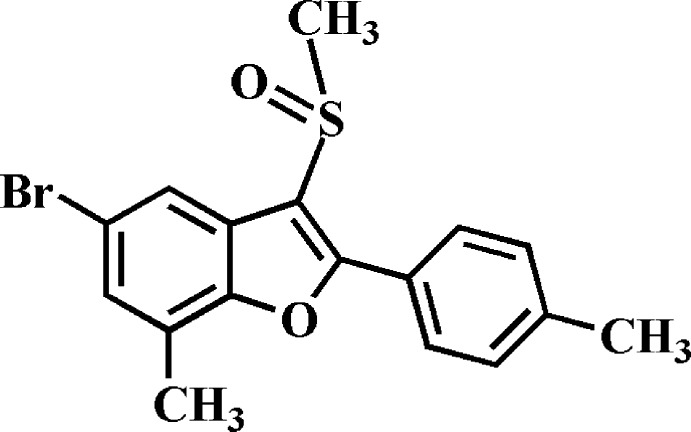


## Experimental

### 

#### Crystal data

C_17_H_15_BrO_2_S
*M* *_r_* = 363.26
Triclinic, 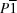

*a* = 7.6375 (2) Å
*b* = 9.5170 (2) Å
*c* = 11.2413 (2) Å
α = 75.513 (1)°
β = 84.848 (1)°
γ = 71.626 (1)°
*V* = 750.72 (3) Å^3^
*Z* = 2
Mo *K*α radiation
μ = 2.88 mm^−1^
*T* = 173 K
0.28 × 0.24 × 0.22 mm

#### Data collection

Bruker SMART APEXII CCD diffractometer
Absorption correction: multi-scan (*SADABS*; Bruker, 2009 ▶) *T*_min_ = 0.504, *T*_max_ = 0.568
14012 measured reflections
3716 independent reflections
3331 reflections with *I* > 2σ(*I*)
*R*_int_ = 0.040

#### Refinement

*R*[*F* ^2^ > 2σ(*F* ^2^)] = 0.028
*wR*(*F* ^2^) = 0.075
*S* = 1.05
3716 reflections
193 parameters
H-atom parameters constrained
Δρ_max_ = 0.48 e Å^−3^
Δρ_min_ = −0.56 e Å^−3^

### 

Data collection: *APEX2* (Bruker, 2009 ▶); cell refinement: *SAINT* (Bruker, 2009 ▶); data reduction: *SAINT*; program(s) used to solve structure: *SHELXS97* (Sheldrick, 2008 ▶); program(s) used to refine structure: *SHELXL97* (Sheldrick, 2008 ▶); molecular graphics: *ORTEP-3* (Farrugia, 2012 ▶) and *DIAMOND* (Brandenburg, 1998 ▶); software used to prepare material for publication: *SHELXL97*.

## Supplementary Material

Crystal structure: contains datablock(s) global, I. DOI: 10.1107/S160053681204682X/rz5021sup1.cif

Structure factors: contains datablock(s) I. DOI: 10.1107/S160053681204682X/rz5021Isup2.hkl

Supplementary material file. DOI: 10.1107/S160053681204682X/rz5021Isup3.cml

Additional supplementary materials:  crystallographic information; 3D view; checkCIF report

## Figures and Tables

**Fig. 1.**
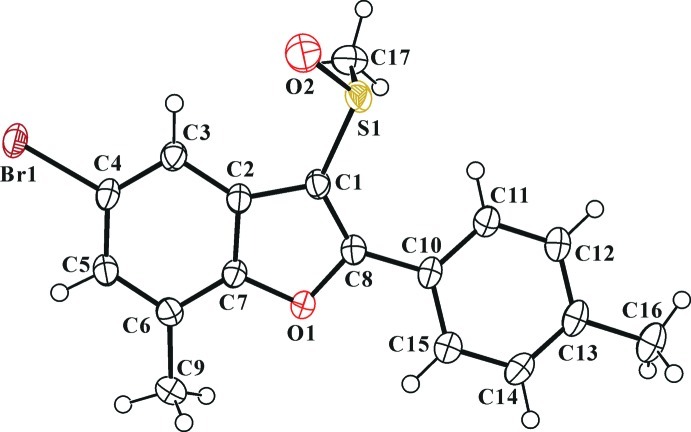
The molecular structure of the title compound with the atom numbering scheme. Displacement ellipsoids are drawn at the 50% probability level. H atoms are presented as small spheres of arbitrary radius.

## supplementary crystallographic information

### Comment

As a part of our ongoing study of 5-bromo-7-methyl-3-methylsulfinyl-1-benzofuran derivatives containing 4-fluorophenyl (Choi *et al.*, 2010) and methyl (Seo *et al.*, 2009) substituents in 2-position, we report herein the crystal structure of the title compound.

In the title molecule (Fig. 1), the benzofuran unit is essentially planar, with a mean deviation of 0.005 (1) Å from the least-squares plane defined by the nine constituent atoms. The dihedral angle between the 4-methylphenyl ring and the mean plane of the benzofuran ring is 14.46 (5)°. In the crystal structure, molecules are linked via pairs of Br···O halogen-bondings between the bromine and the oxygen of the S═O unit [Br1···O2^i^ = 3.151 (2) Å, C4—Br1···O2^i^ = 166.55 (6)°, symmetry code : (i) - *x* + 2, - *y* + 1, - *z* + 2] (Politzer *et al.*, 2007) into centrosymmetric dimers.

### Experimental

3-Chloroperoxybenzoic acid (77%, 224 mg, 1.0 mmol) was added in small portions to a stirred solution of 5-bromo-7-methyl-2-(4-methylphenyl)-3-methylsulfanyl-1-benzofuran (312 mg, 0.9 mmol) in dichloromethane (40 mL) at 273 K. After being stirred at room temperature for 5h, the mixture was washed with saturated sodium bicarbonate solution and the organic layer was separated, dried over magnesium sulfate, filtered and concentrated at reduced pressure. The residue was purified by column chromatography (hexane/ethyl acetate, 1:1 *v*/*v*) to afford the title compound as a colorless solid [yield 68%, m.p. 462–463 K; Rf = 0.53 (hexane/ethyl acetate, 1:1 *v*/*v*)]. Single crystals suitable for X-ray diffraction were prepared by slow evaporation of a solution of the title compound in acetone at room temperature.

### Refinement

All H atoms were positioned geometrically and refined using a riding model, with C—H = 0.95 Å for aryl and 0.98 Å for methyl H atoms, and with *U*_iso_(H) = 1.2*U*_eq_(C) for aryl and 1.5*U*_eq_(C) for methyl H atoms. The positions of methyl hydrogens were optimized rotationally.

### Crystal data

**Table:**

C_17_H_15_BrO_2_S	*Z* = 2
*M**_r_* = 363.26	*F*(000) = 368
Triclinic, *P*1	*D*_x_ = 1.607 Mg m^−^^3^
Hall symbol: -P 1	Melting point = 462–463 K
*a* = 7.6375 (2) Å	Mo *K*α radiation, λ = 0.71073 Å
*b* = 9.5170 (2) Å	Cell parameters from 6280 reflections
*c* = 11.2413 (2) Å	θ = 2.6–28.3°
α = 75.513 (1)°	µ = 2.88 mm^−^^1^
β = 84.848 (1)°	*T* = 173 K
γ = 71.626 (1)°	Block, colourless
*V* = 750.72 (3) Å^3^	0.28 × 0.24 × 0.22 mm

### Data collection

**Table:**

Bruker SMART APEXII CCD diffractometer	3716 independent reflections
Radiation source: rotating anode	3331 reflections with *I* > 2σ(*I*)
Graphite multilayer monochromator	*R*_int_ = 0.040
Detector resolution: 10.0 pixels mm^-1^	θ_max_ = 28.3°, θ_min_ = 1.9°
φ and ω scans	*h* = −10→10
Absorption correction: multi-scan (*SADABS*; Bruker, 2009)	*k* = −12→12
*T*_min_ = 0.504, *T*_max_ = 0.568	*l* = −14→14
14012 measured reflections	

### Refinement

**Table:**

Refinement on *F*^2^	Primary atom site location: structure-invariant direct methods
Least-squares matrix: full	Secondary atom site location: difference Fourier map
*R*[*F*^2^ > 2σ(*F*^2^)] = 0.028	Hydrogen site location: difference Fourier map
*wR*(*F*^2^) = 0.075	H-atom parameters constrained
*S* = 1.05	*w* = 1/[σ^2^(*F*_o_^2^) + (0.039*P*)^2^ + 0.174*P*] where *P* = (*F*_o_^2^ + 2*F*_c_^2^)/3
3716 reflections	(Δ/σ)_max_ = 0.002
193 parameters	Δρ_max_ = 0.48 e Å^−^^3^
0 restraints	Δρ_min_ = −0.56 e Å^−^^3^

### Special details

**Table:**

Geometry. All esds (except the esd in the dihedral angle between two l.s. planes) are estimated using the full covariance matrix. The cell esds are taken into account individually in the estimation of esds in distances, angles and torsion angles; correlations between esds in cell parameters are only used when they are defined by crystal symmetry. An approximate (isotropic) treatment of cell esds is used for estimating esds involving l.s. planes.
Refinement. Refinement of F^2^ against ALL reflections. The weighted R-factor wR and goodness of fit S are based on F^2^, conventional R-factors R are based on F, with F set to zero for negative F^2^. The threshold expression of F^2^ > 2sigma(F^2^) is used only for calculating R-factors(gt) etc. and is not relevant to the choice of reflections for refinement. R-factors based on F^2^ are statistically about twice as large as those based on F, and R- factors based on ALL data will be even larger.

### Fractional atomic coordinates and isotropic or equivalent isotropic displacement parameters (Å^2^)

**Table:**

	*x*	*y*	*z*	*U*_iso_*/*U*_eq_	
Br1	1.01640 (2)	0.25733 (2)	1.044940 (18)	0.03262 (8)	
S1	0.65991 (7)	0.79517 (5)	0.61463 (5)	0.03608 (13)	
O1	0.80729 (16)	0.39887 (14)	0.52060 (12)	0.0266 (3)	
O2	0.7824 (3)	0.80345 (17)	0.70610 (17)	0.0516 (4)	
C1	0.7300 (2)	0.60520 (19)	0.60028 (18)	0.0262 (4)	
C2	0.8166 (2)	0.47429 (19)	0.69618 (17)	0.0247 (3)	
C3	0.8598 (2)	0.44924 (19)	0.81916 (18)	0.0270 (4)	
H3	0.8304	0.5300	0.8601	0.032*	
C6	0.9504 (2)	0.2018 (2)	0.69871 (19)	0.0278 (4)	
C10	0.6566 (2)	0.6248 (2)	0.37333 (17)	0.0258 (3)	
C4	0.9481 (2)	0.3001 (2)	0.87834 (18)	0.0265 (4)	
C14	0.6396 (3)	0.6087 (2)	0.16459 (19)	0.0339 (4)	
H14	0.6760	0.5516	0.1034	0.041*	
C5	0.9929 (2)	0.1788 (2)	0.82077 (18)	0.0285 (4)	
H5	1.0535	0.0787	0.8661	0.034*	
C8	0.7269 (2)	0.55446 (19)	0.49709 (17)	0.0251 (3)	
C7	0.8620 (2)	0.35210 (19)	0.64087 (17)	0.0244 (3)	
C12	0.4659 (3)	0.8359 (2)	0.22513 (19)	0.0322 (4)	
H12	0.3837	0.9368	0.2061	0.039*	
C13	0.5173 (2)	0.7551 (2)	0.13389 (19)	0.0315 (4)	
C11	0.5323 (3)	0.7721 (2)	0.34314 (19)	0.0310 (4)	
H11	0.4931	0.8286	0.4045	0.037*	
C15	0.7097 (3)	0.5440 (2)	0.28117 (19)	0.0306 (4)	
H15	0.7943	0.4441	0.2990	0.037*	
C16	0.4439 (3)	0.8233 (3)	0.0066 (2)	0.0401 (5)	
H16A	0.3579	0.9255	0.0027	0.060*	
H16B	0.3794	0.7591	−0.0149	0.060*	
H16C	0.5464	0.8300	−0.0514	0.060*	
C9	0.9909 (3)	0.0770 (2)	0.6320 (2)	0.0389 (5)	
H9A	1.0309	−0.0220	0.6908	0.058*	
H9B	1.0890	0.0862	0.5708	0.058*	
H9C	0.8793	0.0850	0.5907	0.058*	
C17	0.4442 (3)	0.7945 (3)	0.6906 (2)	0.0482 (6)	
H17A	0.4645	0.7094	0.7633	0.072*	
H17B	0.3613	0.7832	0.6344	0.072*	
H17C	0.3884	0.8906	0.7158	0.072*	

### Atomic displacement parameters (Å^2^)

**Table:**

	*U*^11^	*U*^22^	*U*^33^	*U*^12^	*U*^13^	*U*^23^
Br1	0.04139 (12)	0.03114 (11)	0.02435 (12)	−0.01163 (8)	−0.01237 (8)	0.00023 (8)
S1	0.0551 (3)	0.0203 (2)	0.0333 (3)	−0.01144 (19)	−0.0163 (2)	−0.00163 (19)
O1	0.0316 (6)	0.0258 (6)	0.0205 (7)	−0.0063 (5)	−0.0039 (5)	−0.0037 (5)
O2	0.0753 (11)	0.0340 (8)	0.0538 (11)	−0.0211 (7)	−0.0298 (9)	−0.0090 (7)
C1	0.0327 (8)	0.0213 (7)	0.0247 (10)	−0.0096 (6)	−0.0067 (7)	−0.0014 (7)
C2	0.0276 (8)	0.0232 (8)	0.0234 (10)	−0.0095 (6)	−0.0055 (7)	−0.0016 (7)
C3	0.0328 (8)	0.0251 (8)	0.0247 (10)	−0.0104 (7)	−0.0067 (7)	−0.0042 (7)
C6	0.0272 (8)	0.0256 (8)	0.0288 (10)	−0.0046 (6)	−0.0037 (7)	−0.0062 (7)
C10	0.0283 (8)	0.0292 (8)	0.0202 (9)	−0.0124 (7)	−0.0042 (7)	−0.0004 (7)
C4	0.0289 (8)	0.0295 (8)	0.0211 (9)	−0.0106 (7)	−0.0076 (7)	−0.0013 (7)
C14	0.0405 (10)	0.0418 (10)	0.0217 (10)	−0.0152 (8)	−0.0013 (8)	−0.0078 (8)
C5	0.0294 (8)	0.0248 (8)	0.0283 (10)	−0.0066 (6)	−0.0069 (7)	−0.0008 (7)
C8	0.0261 (8)	0.0243 (8)	0.0243 (10)	−0.0089 (6)	−0.0033 (7)	−0.0018 (7)
C7	0.0260 (8)	0.0261 (8)	0.0209 (9)	−0.0081 (6)	−0.0034 (6)	−0.0038 (7)
C12	0.0337 (9)	0.0334 (9)	0.0261 (11)	−0.0093 (7)	−0.0065 (8)	0.0000 (8)
C13	0.0312 (9)	0.0429 (10)	0.0215 (10)	−0.0172 (8)	−0.0055 (7)	0.0001 (8)
C11	0.0362 (9)	0.0313 (9)	0.0238 (10)	−0.0088 (7)	−0.0048 (7)	−0.0037 (8)
C15	0.0331 (9)	0.0319 (9)	0.0256 (10)	−0.0098 (7)	−0.0019 (7)	−0.0042 (8)
C16	0.0398 (10)	0.0549 (13)	0.0250 (11)	−0.0181 (9)	−0.0075 (8)	−0.0009 (10)
C9	0.0461 (11)	0.0272 (9)	0.0374 (13)	0.0001 (8)	−0.0038 (9)	−0.0103 (9)
C17	0.0543 (13)	0.0416 (12)	0.0459 (15)	−0.0019 (10)	−0.0051 (11)	−0.0202 (11)

### Geometric parameters (Å, º)

**Table:**

Br1—C4	1.8987 (19)	C14—C15	1.377 (3)
Br1—O2^i^	3.1514 (17)	C14—C13	1.389 (3)
S1—O2	1.4825 (16)	C14—H14	0.9500
S1—C1	1.7611 (17)	C5—H5	0.9500
S1—C17	1.787 (3)	C12—C11	1.382 (3)
O1—C7	1.372 (2)	C12—C13	1.389 (3)
O1—C8	1.378 (2)	C12—H12	0.9500
C1—C8	1.368 (3)	C13—C16	1.494 (3)
C1—C2	1.445 (2)	C11—H11	0.9500
C2—C7	1.388 (2)	C15—H15	0.9500
C2—C3	1.395 (3)	C16—H16A	0.9800
C3—C4	1.385 (2)	C16—H16B	0.9800
C3—H3	0.9500	C16—H16C	0.9800
C6—C5	1.386 (3)	C9—H9A	0.9800
C6—C7	1.389 (2)	C9—H9B	0.9800
C6—C9	1.497 (3)	C9—H9C	0.9800
C10—C15	1.398 (3)	C17—H17A	0.9800
C10—C11	1.401 (3)	C17—H17B	0.9800
C10—C8	1.454 (3)	C17—H17C	0.9800
C4—C5	1.398 (3)		
			
C4—Br1—O2^i^	166.55 (6)	O1—C7—C6	123.97 (16)
O2—S1—C1	106.92 (9)	C2—C7—C6	124.96 (18)
O2—S1—C17	106.93 (12)	C11—C12—C13	121.09 (18)
C1—S1—C17	97.14 (10)	C11—C12—H12	119.5
C7—O1—C8	106.78 (13)	C13—C12—H12	119.5
C8—C1—C2	107.54 (15)	C12—C13—C14	117.90 (18)
C8—C1—S1	127.55 (14)	C12—C13—C16	121.17 (18)
C2—C1—S1	124.74 (14)	C14—C13—C16	120.94 (19)
C7—C2—C3	119.56 (16)	C12—C11—C10	120.76 (19)
C7—C2—C1	104.61 (16)	C12—C11—H11	119.6
C3—C2—C1	135.82 (17)	C10—C11—H11	119.6
C4—C3—C2	116.29 (16)	C14—C15—C10	120.27 (18)
C4—C3—H3	121.9	C14—C15—H15	119.9
C2—C3—H3	121.9	C10—C15—H15	119.9
C5—C6—C7	114.80 (16)	C13—C16—H16A	109.5
C5—C6—C9	124.04 (17)	C13—C16—H16B	109.5
C7—C6—C9	121.14 (18)	H16A—C16—H16B	109.5
C15—C10—C11	118.12 (18)	C13—C16—H16C	109.5
C15—C10—C8	120.06 (16)	H16A—C16—H16C	109.5
C11—C10—C8	121.81 (17)	H16B—C16—H16C	109.5
C3—C4—C5	123.18 (18)	C6—C9—H9A	109.5
C3—C4—Br1	118.81 (14)	C6—C9—H9B	109.5
C5—C4—Br1	118.00 (13)	H9A—C9—H9B	109.5
C15—C14—C13	121.86 (19)	C6—C9—H9C	109.5
C15—C14—H14	119.1	H9A—C9—H9C	109.5
C13—C14—H14	119.1	H9B—C9—H9C	109.5
C6—C5—C4	121.20 (16)	S1—C17—H17A	109.5
C6—C5—H5	119.4	S1—C17—H17B	109.5
C4—C5—H5	119.4	H17A—C17—H17B	109.5
C1—C8—O1	109.99 (15)	S1—C17—H17C	109.5
C1—C8—C10	135.25 (16)	H17A—C17—H17C	109.5
O1—C8—C10	114.74 (15)	H17B—C17—H17C	109.5
O1—C7—C2	111.07 (15)		
			
O2—S1—C1—C8	−146.49 (17)	C15—C10—C8—C1	166.89 (19)
C17—S1—C1—C8	103.34 (18)	C11—C10—C8—C1	−14.6 (3)
O2—S1—C1—C2	28.10 (19)	C15—C10—C8—O1	−14.8 (2)
C17—S1—C1—C2	−82.07 (17)	C11—C10—C8—O1	163.75 (15)
C8—C1—C2—C7	0.78 (19)	C8—O1—C7—C2	0.72 (18)
S1—C1—C2—C7	−174.73 (13)	C8—O1—C7—C6	−179.55 (16)
C8—C1—C2—C3	−179.73 (19)	C3—C2—C7—O1	179.48 (14)
S1—C1—C2—C3	4.8 (3)	C1—C2—C7—O1	−0.92 (19)
C7—C2—C3—C4	0.3 (2)	C3—C2—C7—C6	−0.2 (3)
C1—C2—C3—C4	−179.18 (19)	C1—C2—C7—C6	179.35 (16)
C2—C3—C4—C5	−0.2 (3)	C5—C6—C7—O1	−179.58 (15)
C2—C3—C4—Br1	178.66 (12)	C9—C6—C7—O1	−0.8 (3)
O2^i^—Br1—C4—C3	−92.2 (3)	C5—C6—C7—C2	0.1 (3)
O2^i^—Br1—C4—C5	86.6 (3)	C9—C6—C7—C2	178.91 (18)
C7—C6—C5—C4	0.0 (3)	C11—C12—C13—C14	−0.9 (3)
C9—C6—C5—C4	−178.76 (18)	C11—C12—C13—C16	179.26 (18)
C3—C4—C5—C6	0.0 (3)	C15—C14—C13—C12	−0.2 (3)
Br1—C4—C5—C6	−178.79 (13)	C15—C14—C13—C16	179.66 (18)
C2—C1—C8—O1	−0.37 (19)	C13—C12—C11—C10	1.4 (3)
S1—C1—C8—O1	174.97 (13)	C15—C10—C11—C12	−0.8 (3)
C2—C1—C8—C10	178.02 (18)	C8—C10—C11—C12	−179.30 (16)
S1—C1—C8—C10	−6.6 (3)	C13—C14—C15—C10	0.8 (3)
C7—O1—C8—C1	−0.20 (18)	C11—C10—C15—C14	−0.3 (3)
C7—O1—C8—C10	−178.95 (14)	C8—C10—C15—C14	178.27 (16)

Symmetry code: (i) −*x*+2, −*y*+1, −*z*+2.
